# Pleiotropic regulation of bacterial toxin production and Allee effect govern microbial predator–prey interactions

**DOI:** 10.1093/ismeco/ycaf031

**Published:** 2025-02-14

**Authors:** Harikumar R Suma, Pierre Stallforth

**Affiliations:** Department of Paleobiotechnology, Leibniz Institute for Natural Product Research and Infection Biology—Leibniz-HKI, Beutenbergstrasse 11a, 07745 Jena, Germany; Cluster of Excellence Balance of the Microverse, Friedrich Schiller University Jena, Fürstengraben 1, 07743 Jena, Germany; Department of Paleobiotechnology, Leibniz Institute for Natural Product Research and Infection Biology—Leibniz-HKI, Beutenbergstrasse 11a, 07745 Jena, Germany; Faculty of Chemistry and Earth Sciences, Friedrich Schiller University, Humboldtstrasse 10, 07743 Jena, Germany

**Keywords:** pleiotropic regulation, Allee effect, social amoebae, *Dictyostelium discoideum*, *Pseudomonas*, pyreudiones, microbial natural products

## Abstract

Bacteria are social organisms, which are constantly exposed to predation by nematodes or amoebae. To counteract these predation pressures, bacteria have evolved a variety of potent antipredator strategies. Bacteria of the genus *Pseudomonas*, for instance, evade amoebal predation by the secretion of amoebicidal natural products. The soil bacterium *Pseudomonas fluorescens* HKI0770 produces pyreudione alkaloids that can kill amoebae. Even though the mode of action of the pyreudiones has been elucidated, the spatiotemporal dynamics underlying this predator–prey interaction remain unknown. Using a combination of microscopy and analytical techniques, we elucidated the intricate relationship of this predator–prey association. We used the chromatic bacteria toolbox for intraspecific differentiation of the amoebicide-producing wildtype and the non-producing mutant within microcosms. These allow for variations in nutrient availability and the emergence of predation-evasion strategies of interacting microorganisms. Imaging of the co-cultures revealed that the amoebae initially ingest both the non-producer as well as the toxin-producer cells. The outcomes of predator–prey interactions are governed by the population size and fitness of the interacting partners. We identified that changes in the cell density coupled with alterations in nutrient availability led to a strong Allee effect resulting in the diminished production of pyreudione A. The loss of defense capabilities renders *P. fluorescens* HKI0770 palatable to amoebae. Such a multifaceted regulation provides the basis for a model by which predator–prey populations are being regulated in specific niches. Our results demonstrate how the spatiotemporal regulation of bacterial toxin production alters the feeding behavior of amoeba.

## Graphical Abstract

**Graphical Abstract:**
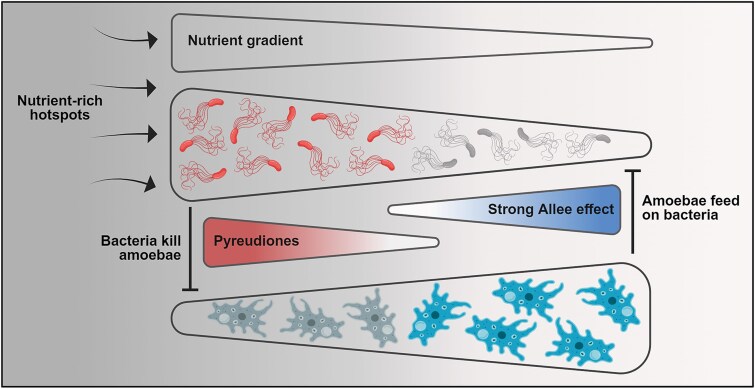

## Introduction

Bacteria engage in a wide range of social behaviors when interacting with other microorganisms of the same or different species [1]. Soil-dwelling bacteria are exposed to various bacterivores, especially protists, which regulate bacterial populations and are crucial in shaping natural microbial communities [2–6]. The abundance and diversity of protists such as amoebae strongly depend on several environmental factors such as nutrient availability, temperature, and moisture [7]. In nutrient-rich hotspots such as the rhizosphere, bacteria utilize dissolved organic matter derived from plants or animals. Subsequently, amoebae, along with other bacterivores feed on these prokaryotes thus facilitating the release of various nutrients into the soil that would otherwise remain sequestered in the bacterial biomass. In doing so, plants are able to mobilize these nutrients, thereby stimulating plant growth [8]. As the plants grow, they secrete more root exudates increasing the bacterial density which in turn benefits the predators. Hence, this microbial loop is thought to ensure the fitness and protection of the interacting partners and facilitate nutrient recycling through microbial interactions [9, 10].

From an ecological standpoint, bacterial predation plays a critical role in maintaining the equilibrium of biomes as it curtails the uncontrolled proliferation of certain bacterial species, promoting microbial diversity, and enhancing nutrient cycling [11–15]. Faced with such strong predation pressure, bacteria have evolved intricate defense mechanisms based on either the creation of physical barriers such as biofilms, [16, 17] increased motility [18, 19] alongside intracellular survival of bacteria within the predator [20–22]. The biosynthesis of toxic low molecular weight natural products, on the other hand, represents a potent defense mechanism to kill predators [23–27]. The co-evolution of microorganisms within polymicrobial associations has even led to the emergence of cooperative antipredator defense strategies [28, 29]. To counteract these defense mechanisms, predators such as amoebae have evolved efficient counterstrategies including lectin agglutination of bacteria and production of antimicrobials [3, 30–33]. As a consequence, these evolutionary arms races have contributed to the emergence of bacterial virulence and diverse cell-autonomous defense mechanisms. Together they highlight the role of microbial predator–prey interactions as an important source of highly adapted natural products with significant clinical and agricultural applications [34–36].

Predation of bacteria by protists can be studied conveniently in the laboratory using the model organism *Dictyostelium discoideum*, a ubiquitous social amoeba. In its vegetative form, the motile amoeboid cells are chemotactically attracted to bacteria, which they feed on by a process known as phagocytosis. Cell division occurs mitotically. When bacterial food sources are depleted, amoebae starve and enter a multicellular sorocarpic life cycle [37]. In this stage, individual cells (ca. 10^5^) aggregate, generating a fruiting body (or sporocarp) consisting of a basal plate, a stalk, and a spore-containing sorus on top [38]. Other dormant stages, such as cysts, may also form in response to various biotic and abiotic factors across the *Dictyostelium* taxon groups [39, 40]. Since *D. discoideum* exhibits an intricate relationship with its interacting partners, it is, therefore, an excellent platform for studying a wide range of eukaryote–bacteria interactions spanning the whole spectrum of symbiosis—from mutualism to antagonism [22, 41, 42].

An example of one such interspecies interaction is that of amoebae and the bacterium *Pseudomonas fluorescens* HKI0770. *P. fluorescens* HKI0770 is a soil bacterium known to secrete extracellular amoebicidal natural products—pyreudiones (pys)—enabling the bacteria to evade predation [23]. The mutant strain unable to produce pyreudiones becomes palatable to amoebae, further emphasizing the role of small molecules in shaping predator–prey interactions. The constitutive expression of pyreudiones and the ability of pyreudiones to induce intralysosomal acidification in amoebae effectively turn the bacterium into an extracellular pathogen to amoebae [43]. Hence, this system is an excellent model for studying antagonistic microbial predator–prey interactions, further highlighting the significance of elucidating the underlying dynamics of the relationship between *P. fluorescens* HKI0770 and amoebae.

A key factor in determining the dynamics of such predator–prey interactions is the relationship between population density and fitness of both predator and prey [44–46]. Such population-dependent outcomes between amoebae and bacteria are known as the Allee effect [47–50]. Allee effects, in general, are population-dependent processes that are relevant for social and cooperative organisms and occur as a result of inter- or intraspecific interactions among microorganisms [51–54]. In particular, a strong Allee effect arises when the growth rate of an organism declines below a certain threshold such that the same organism is unable to defend itself from the detrimental effects of another interacting organism. Eventually creating a negative impact on the per capita growth of the smaller population [55].

As social organisms, amoebae, and bacteria are influenced by population-dependent processes such as the Allee effect due to interspecific or intraspecific interactions and changes in environmental conditions [47, 52, 56, 57]. Access to nutrient-rich hotspots for instance the rhizosphere, provides a selective advantage for bacteria enabling near-exponentially growth to compensate for predation losses. However, alterations in environmental conditions such as nutrient depletion can intensify deleterious factors such as grazing pressure [58]. Even though the secretion of amoebicidal natural products is a potent anti-predator strategy, the biosynthesis of these molecules involves diverting resources from primary metabolism posing an energy burden to the individual cell [59, 60]. Hence, the regulation of natural product biosynthesis often depends on environmental factors such as nutrient availability or cell density [61]. Quorum sensing – a mechanism by which the accumulation of bacterially produced small molecules is sensed - allows bacteria to coordinate population-dependent behaviors. These can include the production of virulence factors, biofilm formation, as well as the secretion of toxic small molecules [62].

While amoebal predation is evaded by the bacterium’s production of the amoebicide pyreudione, it remains unknown how the amoebae and bacteria interact on a microscopic scale and—for instance—if the bacteria are actually ingested. Since pyreudione production is not mediated by quorum sensing and considering the multifaceted nature of ecological factors shaping predator–prey interactions, we thus hypothesized the involvement of pleiotropic or other population-dependent factors that could influence the production of pyreudione A [56]. Studies have shown that the outcomes of amoebae−bacteria interactions are strongly affected by cell density and nutrient availability, but each parameter has been studied independently [42, 47, 63]. We also proposed the hypothesis that a non-cell autonomous regulation can alter the feeding behavior of amoebae and modulate the dynamics of the predator–prey interaction between *D. discoideum* and *P. fluorescens* HKI0770. Additionally, the effect of these environmental factors on the existing microbial loop concept also remains poorly understood. To understand the influence of non-cell autonomous factors on the regulation of toxic natural products, we examined the dynamics of predator–prey interaction between *D. discoideum* and *P. fluorescens* HKI0770 in detail.

## Material and methods

### Dictyostelium discoideum

*D. discoideum* strain AX2 strain was grown in HL5 medium (Formedium, Norfolk, England) supplemented with 1% (w/v) glucose (Carl Roth, Karlsruhe, Germany). *D. discoideum* strain AX3 VatMpr/[act15]:vatM:GFP (hereafter *D. discoideum* vatM:GFP; dictyBase accession no. DBS0237042) strain was grown in HL5 medium (14 g L^−1^ peptone, 13.5 g L^−1^ glucose, 7 g L^−1^ yeast extract, 0.5 g L^−1^ KH_2_PO_4_ and 0.5 g L^−1^ Na_2_HPO_4_) supplemented with 1% (w/v) glucose and 50 μg ml^−1^ geneticin (G418, InvivoGen, Toulouse, France). Both strains were grown at 22°C in cell culture dishes (Sarstedt, Nümbrecht, Germany) or on a gyratory shaker (ISF1-X, Kuhner, Birsfelden, Switzerland) as shaking cultures at 140 rpm.

### *Pseudomonas fluorescens* HKI0770

All *P. fluorescens* HKI0770 strains (wildtype (wt), *∆pys* and chromatic mutants) were cultured at 28°C on a gyratory shaker at 180 rpm in SM/5 broth (Formedium, Norfolk, England), unless mentioned otherwise. Before an experiment, the cells from overnight cultures were harvested by centrifugation (6000 × *g* for 5 min) and washed twice in 1× Sörensen’s (Sor) buffer (pH 6.0; 2 g L^−1^ KH_2_PO_4_ [Carl Roth, Karlsruhe, Germany]), 0.29 g L^−1^ Na_2_HPO_4_ (Carl Roth, Karlsruhe, Germany).

### Chromosomal insertion of fluorescent proteins

The chromatic bacteria toolbox [64] was implemented for the chromosomal integration of fluorescent tags into *P. fluorescens* wt and *∆pys* strains. The plasmids, pMRE-Tn7–140 (encoding for mTagBFP2), and pMRE-Tn7–145 (encoding for mScarlet-I) were obtained from Prof. Dr. Mitja Remus-Emsermann (Freie Universität Berlin, Germany).

The *E. coli* BW29427 strain (an auxotroph of Diaminopimelic acid (DAP)) harboring pMRE-Tn7 plasmids was used as the donor strain. The plasmids, pMRE-Tn7–145, and pMRE-Tn7–140 were delivered into *P. fluorescens* wt and *∆pys* strains respectively. Conjugation was carried out in a ratio of 4:1 (donor:recipient) and drop-spotted on Luria-Bertani (LB) agar (Carl Roth, Karlsruhe, Germany) supplemented with 0.3 mM DAP (Alfa Aesar, MA, USA) and 100 μg ml^−1^ ampicillin (Carl Roth, Karlsruhe, Germany) [65]. For the selection of transformants, the bacterial mix was resuspended on LB agar supplemented with 15 μg ml^−1^ gentamicin (Carl Roth, Karlsruhe, Germany). Chromosomal integration of fluorescent tags was confirmed by colony PCR. The primer pair Fwd_full series_Tn7 and Rev_Tn5/7_gt (Table S1) were used to confirm the integration of fluorescent tags. The primer pair Fwd-Tn7_back and Rev-Tn7_back (Table S1) was used to confirm the absence of the delivery plasmid.

### Plaque assays with amoeba

#### Amoebicidal activity of *Pseudomonas fluorescens* HKI0770 chromatic mutants

The amoebicidal activity of the chromatic mutants were verified using a 24-well plaque assay as previously described [28]. The assay was performed on SM/5 agar plugs present in each well of a 24-well plate (Sarstedt, Nümbrecht, Germany). Overnight cultures of *P. fluorescens* wt, *∆pys,* and their corresponding chromatic mutants were prepared. To the individual wells, 30 μl of the respective bacteria was added and the plate was kept for air drying.

#### Influence of pyreudione A on amoebal predation

The influence of pyreudione A on amoebal predation was investigated by another plaque assay involving the supplementation of different concentrations of pyreudione A (Isolation of pyreudione A). Overnight cultures of *P. fluorescens ∆pys* strains were prepared. To the individual wells,

100 μl of 1× Sor buffer (with 1% DMSO) containing bacterial culture along and the specified concentration of pyreudione A (from 16 μg ml^−1^ down to 0.03 μg ml^−1^) was added to each well and the 24-well plate was kept for air drying. The co-culture of AX2 and *∆pys* strain without pyreudione supplementation was used as a control.

#### Influence of the Allee effect on amoebal predation

The influence of the Allee effect on amoebal predation was tested using another plaque assay. The assay was performed on Peptone Yeast Glucose (hereafter PYG100; 20 g L^−1^ proteose peptone (Carl Roth, Karlsruhe, Germany), 18 g L^−1^ glucose, 2 g L^−1^ yeast extract (Carl Roth, Karlsruhe, Germany) in PAS), 10% of PYG medium (hereafter PYG10) and Page’s Amoeba Saline (hereafter PAS; ATCC medium 1323) agar plugs present in a 24-well plate. Cultures of *P. fluorescens* wt and *∆pys* strains were prepared and the OD_600_ was adjusted to 0.1. Bacterial cells were added to individual wells of each media at a multiplicity of infection (MOI) of 5 or 100 bacteria per amoeba. *Klebsiella aerogenes* (*Ka*), a typical food bacterium for *D. discoideum* was used as a positive control (Fig. S1A).

For all the plaque assays mentioned above*,* ca. 50 000 AX2 cells were added to all the wells. The plates were incubated at 22°C until fruiting body formation occurs. Plaques generated as a result of fruiting body formation indicate that the bacteria are palatable and an absence of plaques indicates the amoebicidal phenotype. The images were acquired using a stereo zoom microscope (Axio Zoom.V16, Carl Zeiss, Jena, Germany) equipped with a PlanApo Z 0.5x/0.125 FWD 114 mm objective lens (Carl Zeiss). The images were processed using ZEN 2.6 (Blue edition, Carl Zeiss) imaging software.

### Isolation and detection of pyreudiones

#### Isolation of pyreudione A

The isolation of pyreudione A was carried out as previously described [23]. For heterologous expression, the *Pseudomonas protegens* Pf-5 *∆gacA* strain [65] harboring the *pys* gene in an arabinose-inducible vector pMQ72 (hereafter *P. protegens* Pf-5 *∆gacA* pMQ72::*pys*) was used [66]. Overnight culture (60 ml) of *P. protegens* Pf-5 *∆gacA* pMQ72::*pys* was prepared in LB liquid medium supplemented with 50 μg ml^−1^ gentamicin and was used to inoculate 3 L of 10x modified Davis media [24] supplemented with 50 μg ml^−1^ gentamicin. At an OD_600_ of 0.6, expression was induced using 100 mM L-arabinose (Carl Roth, Karlsruhe, Germany). The supernatant of the induced culture was extracted with ethyl acetate after 72 h of growth at 28°C on a gyratory shaker at 180 rpm. The crude extract was fractionated using the Isolera™ Prime flash purification system (Biotage, Uppsala, Sweden) equipped with a reverse-phase Sfär C18 D column (100 Å, 30 μm, 30 g, Biotage). A stepwise ACN-water gradient (15, 50, 75, 100% v/v ACN in water) was used and pyreudione A was detected in the 75% ACN fractions by UHPLC–MS analysis. The respective fractions were pooled and the solvents were evaporated for subsequent purification by HPLC. The 75% ACN fraction was dissolved in methanol and filtered using a 0.2 μm PTFE filter. Further purification was carried out using a preparative HPLC system (Shimadzu) equipped with a reverse-phase Luna® C18 column (250 × 21.2 mm, 5 μm, 100 Å, Phenomenex®) and a flow rate of 20 ml min^−1^. The 75% ACN fraction was purified by applying of elution gradient of ACN and water (A: ACN + 0.1% FA, B: Water +0.1% FA; 0–12 min 75% A, 12–14 min linear 75%–100% A) to yield 10 mg (3.3 mg L^−1^) pyreudione A (t_R_ = 11 min).

To determine the calibration curve, the purified pyreudione A was resuspended in 100% DMSO to yield a concentration of 20 mg ml^−1^. From this stock solution, standards (0.03, 0.06, 0.125, 0.25, 0.5, 1.0, 2.0, 4.0, 8.0, 16.0, 32.0 μg ml^−1^) were prepared for the calibration curve. Aliquots from each standard were added to 15 ml of PYG100 media. After 15 min incubation, the media was extracted and samples were prepared for UHPLC–MS as described for the co-culture samples (Time − lapse detection of pyreudione in co-culture). The peak area of pyreudione A (between the retention time of 5.5 and 5.7 min) at λ = 190 nm was extracted for each time point. The concentration of pyreudione A in the co-cultures was determined using the regression equation: Y = 411816*X – 169 932. The standard curve was prepared using Prism software.

#### Detection of pyreudiones using ultra-high performance liquid chromatography–mass spectrometry

The production of pyreudiones by the chromatic mutant as well as the production of pyreudiones by *P. fluorescens* wt in different media (PYG100, PYG10, and PAS) was analysed using Ultra-performance liquid chromatography−mass spectrometry (UHPLC–MS). Overnight cultures (5 ml) were extracted with 10 ml ethyl acetate (VWR, Darmstadt, Germany) and the organic phases were evaporated. The crude extracts were dissolved in 200 μl methanol (VWR, Darmstadt, Germany) and filtered through a 0.2 μm PTFE syringe filter (Sarstedt, Nümbrecht, Germany). UHPLC analyses were carried out using the LCMS-2020 system (Shimadzu, Kyoto, Japan) equipped with a reverse-phase Kinetex® C18 column (250 × 4.6 mm, 5 μm, 100 Å, Phenomenex®, CA, USA). Using a flow rate of 0.7 ml min^−1^, peak separation was obtained with a linear gradient of acetonitrile (ACN; VWR, Darmstadt, Germany) in water supplemented with 0.1% (v/v) formic acid (FA; Sigma−Aldrich/Merck, Darmstadt, Germany). The results were analyzed using LabSolutions 5.6 software (Shimadzu Corporation). The total ion chromatograms (TIC) at the absorbance (λ) of 190 nm were extracted and stacked chromatograms were prepared by Prism 10 (GraphPad) software.

The production titer of pyreudione A in different media (PYG100, PYG10, and PAS) was also examined. Overnight culture of *P. fluorescens* wt prepared in LB liquid medium at 28°C on a gyratory shaker at 180 rpm was used to prepare 5 ml cultures in the three respective media with a starting OD_600_ of 0.5. These overnight cultures were further extracted using ethyl acetate and subjected to UHPLC–MS as mentioned above. The area under the curve (peak area) of pyreudione A, between the retention time (t_R_) of 5.5 and 5.7 min at λ = 190 nm was extracted for each media tested.

#### Time−lapse detection of pyreudione in co-culture

Co-cultures of AX2 and *P. fluorescens* wt were set up in ø9 cm petri dishes (Sarstedt, Nümbrecht, Germany) with PYG100 and PYG10 liquid media. AX2 cells (ca. 2 × 10^6^) were seeded in the dishes and the bacteria were added at a MOI of 5 and 100 per amoeba and incubated at 22°C. As a control, monocultures of *P. fluorescens* wt were also prepared in both media. At the indicated time points (6 h, 12 h, 24 h, and 30 h), the co-cultures (15 ml) were mixed thoroughly and extracted using 30 ml ethyl acetate. After UHPLC analysis, the peak area of pyreudione A (between the retention time of 5.5 and 5.7 min) at λ = 190 nm was extracted for each time point.

### Fluorescence imaging

Imaging was performed using a spinning disk confocal laser scanning microscope (AxioObserver.Z1/7, Carl Zeiss) equipped with a 100x/1.40 NA Plan-Apochromat oil-immersion DIC M27 objective lens (Carl Zeiss). Images were captured using the diode lasers: 405 nm (50 mW), 445 nm (40 mW), 488 nm (50 mW), and 561 nm (50 mW) with the corresponding emission bandpass (BP) filters: BP 450/50, BP 485/30, and BP 629/62.

#### Differentiation of chromatic strains and fluorescence *in situ* hybridization

Overnight cultures of *P. fluorescens* HKI0770 chromatic mutants were prepared. These were resuspended in SM/5 liquid medium and grown again to attain an OD_600_ of 0.5. The cells were harvested and washed twice with 1× Sor buffer. The chromatic mutants were mixed in different ratios (1:1, 1:3, 3:1) and the mixtures were fixed using 4% paraformaldehyde (Sigma−Aldrich/Merck, Darmstadt, Germany) for 4 h at 4°C.

FISH was performed using the probe, PSE227 (probeBase accession no. pB-2539) labeled with ATTO 465 dye (Eurofins Genomics, Ebersberg, Germany). The protocol performed was modified from [67]. A 10 μl aliquot from the various ratios was spread on a microscope slide with wells (Paul Marienfeld, Lauda-Königshofen, Germany). After air drying, sequential ethanol dehydration (50%, 80%, and 100%) was performed. The slide was then incubated with hybridization buffer (1 pmol ml^−1^ PSE227 probe, 20 mM Tris–HCl (Carl Roth, Karlsruhe, Germany), 0.9 M NaCl (Carl Roth, Karlsruhe, Germany), 0.01% SDS (Carl Roth, Karlsruhe, Germany), and 35% formamide (Carl Roth, Karlsruhe, Germany), pH 7.5) at 46°C for 2.5 h. After hybridization, the slide was placed in pre-warmed wash buffer (20 mM Tris–HCl, 0.7 M NaCl, 5 mM Na_2_EDTA (Carl Roth, Karlsruhe, Germany)) for 20 min at 48°C. The slide was then rinsed with ice-cold water. After drying, the cells were mounted in ProLong™ Glass antifade reagent (ThermoFisher Scientific, MA, USA) and a coverslip was placed over the slide.

#### Phagocytosis assay

Live-cell imaging was used to quantify the uptake of the *P. fluorescens* HKI0770 strains by amoebae. Before imaging, ca. 1 × 10^5^  *D. discoideum* vatM:GFP cells were seeded in an 8-well μ-Slide (ibidi, Gräfelfing, Germany) containing 300 μl of HL5 medium. After an hour of incubation, the media was replaced with 1× Sor buffer. Overnight cultures of the *P. fluorescens* HKI0770 chromatic mutants were prepared and resuspended in 1× Sor buffer to an OD_600_ of 0.1. Bipartite co-cultures of chromatic mutants with amoebae were prepared at a MOI of 300 bacteria per amoeba. Tripartite co-cultures were prepared using different ratios (1:1, 1:3, and 3:1) of the chromatic mutants with an overall MOI of 300. The slide was kept in the dark at 22°C for 30 min before imaging. The images were captured as z-stacks with 9–15 optical sections (1 μm per section) and 2 × 2-pixel binning.

The ingested fluorescent bacteria inside an amoebal phagosome were considered as “engulfed bacteria”. This was further represented as the Phagocytic index (PI) [68, 69] calculated according to the formula:


\begin{align*} \mathrm{PI} = & \left(\frac{\mathrm{total}\ \mathrm{no}.\mathrm{of}\ \mathrm{bacteria}\ \mathrm{engulfed}}{\mathrm{total}\ \mathrm{no}.\mathrm{of}\ \mathrm{amoeba}\mathrm{e}\ }\right) \\& \times\left(\frac{\mathrm{total}\ \mathrm{no}.\mathrm{of}\ \mathrm{amoeba}\mathrm{e}\ \mathrm{with}\ \mathrm{engulfed}\ \mathrm{bacteria}}{\mathrm{total}\ \mathrm{no}.\mathrm{of}\ \mathrm{amoeba}\ }\right) \times 100 \end{align*}

### Image analysis

After the acquisition, the images for differentiation of the chromatic mutants were subjected to deblurring (strength-0.3, blur radius-10, and sharpness-0.1) in the ZEN Blue software to improve their clarity. The images of bacteria from various ratios were imported into a CellProfiler [70] pipeline having three “RunCellpose” (Cellpose 1.0.2) modules to segment and count cells in three different channels. The pretrained “bact_fluor_omni” detection network with an expected object diameter of 15, a flow threshold of standard 0.4 and a cell probability threshold of 0.2 was used. A total of 10 images from each ratio were used for the analyses.

The z-stack images from the phagocytosis assay were also subjected to deblurring (strength-0.4, blur radius-50, and sharpness-0.1) in the ZEN Blue software. Two-dimensional maximum projections of z-stacks were obtained using ImageJ 1.54f (Fiji) software. A total of 10 images from the bipartite and tripartite co-cultures were used for the quantification.

### Fold change of amoebae and bacterial population

The co-cultures were prepared using PYG100 and PYG10 medium in ø9cm petri dishes as mentioned above. *P. fluorescens* wt was added to the co-culture at a MOI of 5 and 100 and the dishes were kept at 22°C. To determine the bacterial CFU count (CFU ml^−1^), the supernatant from the co-culture was collected and serial dilutions were carried out. A 100 μl aliquot from the final dilution was plated on LB agar and the resulting colonies were counted.

In order to obtain the AX2 cell numbers, 1× Sor buffer was added to the petri dishes after the supernatant was removed. The adherent amoebae cells were scrapped out and mixed thoroughly. The cell numbers were determined by the fluidlab cell counter (R-300, anvajo, Dresden, Germany).

Fold change is considered as a representative of the fitness of a population [48]. Hence, the fold change of the population (bacteria or amoeba) for each time point was calculated according to the formula:


$$ \mathrm{Fold}\ \mathrm{change}=\frac{\mathrm{cell}\ \mathrm{density}\ \mathrm{at}\ \mathrm{the}\ \mathrm{particular}\ \mathrm{time}\ \mathrm{point}}{\mathrm{cell}\ \mathrm{density}\ \mathrm{at}\ \mathrm{the}\ \mathrm{previous}\ \mathrm{time}\ \mathrm{point}} $$

### Setting up microcosm experiments

#### Dual–agar microcosm

To better understand how the Allee effect and nutrient availability affect the predator–prey relationship, a plaque assay was performed on a microcosm having two parts—one nutrient-rich and one nutrient-poor media.

To set up this system, 6 ml of PYG100 agar was poured into the wells of quadriPERM® tissue culture dish (Sarstedt, Nümbrecht, Germany). Plastic inserts placed at the center of the wells helped to create two halves of the microcosm. Once that agar was solidified, the insert was removed and an equal volume of PYG10 agar was poured from the other side to create a dual–agar microcosm. A similar microcosm was also prepared using PYG10 and PAS media.

The microcosms were inoculated with AX2 cells (ca. 1 × 10^5^) along with *P. fluorescens* wt or the chromatic mutant at a MOI of 5. The amoeba–bacteria co-culture was spread throughout the wells using an inoculation loop (Sarstedt, Nümbrecht, Germany). Once dried, the microcosms were incubated at 22°C. Images were acquired using an EOS 800D camera (Canon, Tokyo, Japan) and a stereo-zoom microscope when fruiting body formation occurs. Fluorescence images of the tissue culture dishes were captured using iBright™ CL1500 Imaging System (Invitrogen™, MA, USA) equipped with a white Epi-LED source and a neutral density excitation filter (400–700 nm).

In order to examine the distribution of pyreudione A in both microcosms, a 1 cm × 1 cm agar piece was obtained from the center (overlap of both media) and from both sides of the microcosm. The agar pieces were mixed thoroughly in ethyl acetate (10 ml) and subjected to sonication for 15 min. Extraction and UHPLC–MS were carried as mentioned above. The extracted-ion chromatogram (EIC) of pyreudione A (m/z 266) from each part of the microcosm was extracted using LabSolutions software and used for comparison.

#### Soil–alginate hydrogel microcosm

To further extend this predator–prey relationship into a more natural condition, a plaque assay was performed on a microcosm composed of soil and alginate beads.

Alginate beads were prepared as previously described [71, 72]. Sterile 1.2% sodium alginate (Sigma−Aldrich/Merck, Darmstadt, Germany) solutions were prepared using PYG100, PYG10, and PAS liquid media. The homogenized alginate solutions were transferred to 20 ml disposable syringes (Henke Sass Wolf, Tuttlingen, Germany) attached to a 20 G needle (B. Braun, Melsungen, Germany). The alginate solutions were added drop-wise from a height of 8 cm into 100 mM CaCl_2_ solution (Carl Roth, Karlsruhe, Germany) while being stirred on a magnetic stirrer (IKA-Werke, Staufen, Germany) at 500 rpm; such that beads with a diameter between 1.8–2.5 mm was formed. The beads were left in the calcium chloride solution for 30 min to complete the gelation process. The beads were harvested by aseptic filtration through Whatman® No.1 filter paper (Sigma−Aldrich/Merck, Darmstadt, Germany) and washed twice with 1× Sor buffer. After draining, the beads were used for microcosm preparation.

Autoclaved soil (pH 6.5, organic matter 23%; Gebr. Mayer Produktions- und Vertriebsgesellschaft, Wahrenholz, Germany) was used as a substrate to set up the microcosm. Portions of soil (8 g) were moistened by adding 3 ml of 1× Sor buffer to facilitate the movement of amoeba inside the soil [73]. These soil portions were inoculated with ca. 5 × 10^5^ amoebae g^−1^ dry weight. *P. fluorescens* wt was subsequently added to the portions at a MOI of 5. Following the inoculation, the soil was thoroughly mixed and added to the wells of a quadriPERM® tissue culture dish. Alginate beads (5 g) made using PYG100 and PYG10 media were carefully placed on either end of a well to create two halves of the microcosm. A similar microcosm was also prepared using alginate beads made using PYG10 and PAS media. The microcosms were incubated at 22°C. Images were acquired using a Canon EOS 800D camera and a stereo-zoom microscope when fruiting body formation occurs.

### Growth of bacteria in amoeba-conditioned medium

Amoeba-conditioned media (ACM) was prepared using PYG100 and PYG10 media as described previously [74]. Briefly, AX2 cells (ca. 1 × 10^6^ cell ml^−1^) were inoculated in PYG100 or PYG10 media and grown under shaking conditions at 22°C for 18–20 h. After incubation, AX2 cells were harvested by centrifugation (500 × *g* for 10 min) and the ACM was further made cell-free by passing through a 0.2 μm cellulose-acetate syringe filter (Sarstedt, Nümbrecht, Germany). For the growth assay, 50% ACM (a 50/50 mix of the ACM and the respective culture media) was used.

Overnight cultures of *P. fluorescens* wt and *∆pys* strains were prepared and OD_600_ of these cultures was adjusted to 1. A volume of 20 μl was transferred to a 96-well plate with a lid (Sarstedt, Nümbrecht, Germany), followed by the addition of the respective ACM (180 μl) making a total volume of 200 μl. The OD_600_ measurements were acquired using an Infinite® 200 PRO microplate reader (Tecan Group, Männedorf, Switzerland) every 15 min for 48 h. As a control, the strains were inoculated in normal PYG100 and PYG10 media.

### Statistical analysis

Prism (GraphPad) software was used to prepare the graphs and perform statistical analysis. Data shown in all the graphs are mean ± standard error. To define significance, a P value of less than 0.05 was used. Asterisks (^*^) are used in the figures to indicate different significance levels and are defined as follows: *P* ≤ 0.05 (^*^), *P* ≤ 0.01 (^*^^*^), *P* ≤ 0.001 (^*^^*^^*^). Wherever a test of normality was required, Shapiro–Wilk’s test (α = 0.05) was performed.

The cell counting experiment with *P. fluorescens* HKI0770 chromatic mutants was analyzed using a two-way analysis of variance (ANOVA) with Holm–Šídák’s multiple comparison test. Analysis between the phagocytic indices of wt and *∆pys* strains in bipartite co-culture was performed using Mann–Whitney test. Statistical analyses of phagocytic indices among different ratios (inter-ratio comparison) were performed with two-way ANOVA with Holm–Šídák’s multiple comparison test. The experiments involving timeline data (production of pyreudione A and fold change amoeba and bacteria) were analyzed using a two-way ANOVA with Holm–Šídák’s multiple comparison test at each time point.

## Results

### Fluorescent labels allow the differentiation of *Pseudomonas fluorescens* HKI0770 strains

In order to enable the differentiation of *P. fluorescens* HKI0770 wildtype (wt) and the *∆pys* mutant, deficient in the production of amoebicidal pyreudiones, we used the chromatic bacteria toolbox [64] as a differential labeling approach. To this end, we chromosomally inserted different fluorescent tags into the genome of each bacterial strain. Specifically, *P. fluorescens* HKI0770 was labeled with mScarlet-I (wt::MRE-145) and *P. fluorescens* HKI0770 *∆pys* was labeled with mTagBFP2 (*∆pys*::MRE-140).

**Figure 1:**
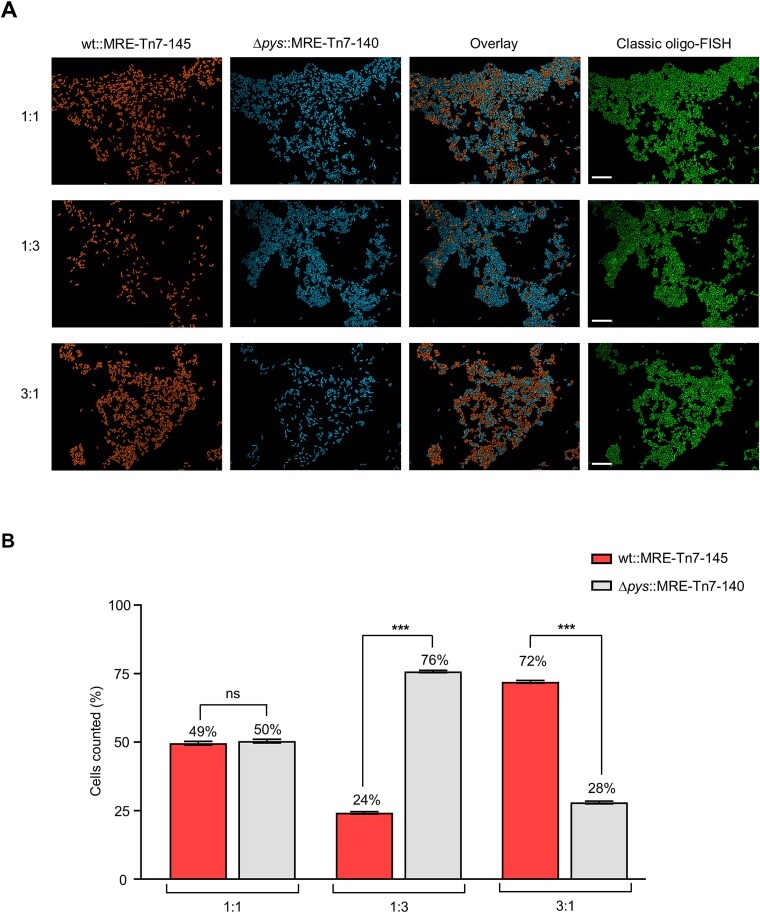
Discrimination between *P. fluorescens* HKI0770 strains using fluorescence imaging. (A) Representative fluorescence microscopy images of *P. fluorescens* wt::MRE-145 expressing mScarlet-I (red) and *P. fluorescens ∆pys*::MRE-140 expressing mTagBFP2 (blue). Different ratios (1:1, 1:3, 3:1) of both strains were used for differentiation. The corresponding FISH image (green) using the PSE227 probe is on the right side. (B) Cell counting using CellProfiler software with different ratios of *P. fluorescens* HKI0770 chromatic mutants. Data shown are mean ± standard error (*n* = 10 images per ratio). Graphs are representative of three independent experiments. The statistical significance was calculated by a two-way ANOVA with Holm–Šídák’s multiple-comparisons test (symbols: ns not significant; ^*^^*^^*^P ≤ 0.001. Scale bars: 10 μm.

For the discrimination of these labeled strains within co-cultures, fluorescence microscopy was performed across different ratios (1:1, 1:3, 3:1) of the individual strains. The differentially tagged cell populations could be unambiguously distinguished using this approach (Fig. 1A). Fluorescence emitted by both bacteria was detected with single-cell resolution facilitating the differentiation even within mixtures. This further helped in the quantification of bacterial cell numbers from different initial cell ratios (Fig. 1B). In the 1:1 ratio, the relative difference between the percentages of wt::MRE-145 and *∆pys*::MRE-140 cells was a factor of 1 (*P* = 0.39). For 1:3 and 3:1 ratios, the relative difference between the percentages of wt::MRE-145 and *∆pys*::MRE-140 cells was a factor of 3 (*P* ≤ 0.001 for 1:3 and 3:1). Further indicating that the percentage of bacteria remained proportional to the initial inoculation ratios. These ratios are used in subsequent co-culture experiments with amoebae.

Importantly, the phenotypes of the chromatic mutants, such as the production of pyreudiones and the feeding behavior of amoeba, remained unaffected by the labeling (Fig. S1).

### Amoeba can ingest the pyreudione−producing bacteria

During co-culture, the ability of amoebae to ingest toxin producers in the absence of pyreudiones remains unknown. To investigate this aspect, we prepared co-cultures of *P. fluorescens* HKI0770 chromatic mutants with *D. discoideum* cells expressing GFP-fused phagosomes. The co-cultures consisted of bipartite and tripartite combinations of chromatic mutants with amoebae.

**Figure 2:**
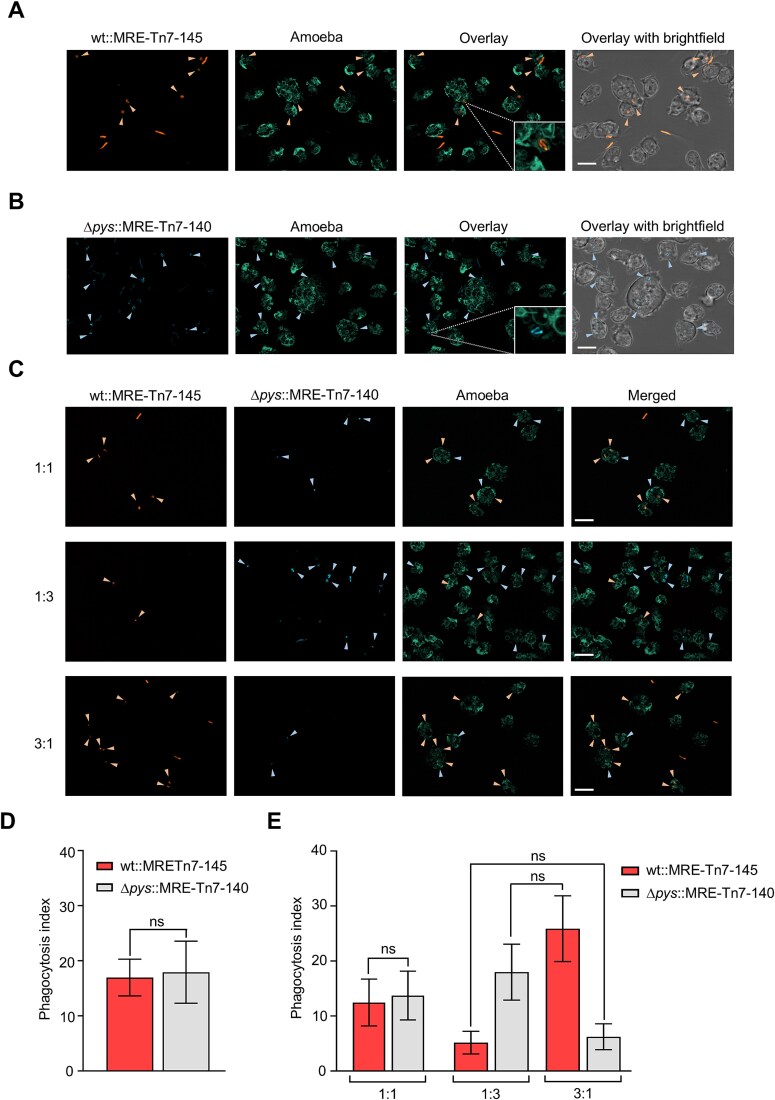
Phagocytosis of *P. fluorescens* HKI0770 strains by amoebae. (A) A representative 2D maximum projection of the bipartite co-culture between the amoeba, *D. discoideum* vatM:GFP and *P. fluorescens* wt::MRE-145 (orange). (B) A representative 2D maximum projection of the bipartite co-culture of amoeba and *P. fluorescens ∆pys*::MRE-140 (blue). The insets show engulfed bacteria (orange or blue) inside fluorescent phagosomes indicating the amoeba can ingest both the producer strain and the mutant. (C) Representative 2D maximum projections of tripartite co-cultures between amoeba and different ratios of wt and *∆pys* cells. Orange and blue arrow heads indicate wt and *∆pys* cells inside phagosomes respectively. (D) Comparison of the phagocytic indices between wt and *∆pys* strains in bipartite co-cultures with amoebae. Statistical significance between the phagocytic indices of wt and *∆pys* strains was calculated by Mann–Whitney test (E) Comparison of the phagocytic indices in tripartite co-cultures among different ratios of wt and *∆pys* cells. Inter-ratio comparisons of the phagocytosis indices (between 1:3 and 3:1 ratios) were carried out using a two-way ANOVA with Holm–Šídák’s multiple comparisons test. Amoebae can ingest both strains and do not exhibit any preferential feeding behavior. Both (D) and (E) were performed in three independent experiments. Graphs are representative of three independent experiments. Data shown are mean ± standard error (*n* = 10 images per ratio). (symbols: ns not significant). Scale bars are 10 μm.

Live-cell imaging of the co-cultures revealed that *P. fluorescens* wt was indeed ingested by *D. discoideum* cells. The bacteria were observed to be engulfed within the fluorescent phagosomes of the amoebae (Fig. 2A). *P. fluorescens ∆pys*, which has been already shown to be palatable to amoebae was used as an edible control (Fig. 2B). The efficacy of phagocytosis was determined by calculating the phagocytic index (PI) of amoebae. The phagocytic index provides a quantitative measure of phagocytic activity, accounting for both the average number of particles engulfed per amoebae and the ratio of amoebae performing phagocytosis [68, 69, 75]. In the bipartite co-culture (Fig. 2D), no significant differences (*P* = 0.68) between the PIs of *P. fluorescens* wt (16.9 ± 3.3) and *∆pys* (17.9 ± 5.6) were observed. This indicates that the amoeba ingested both the amoebicide producer and the non-producer.

In the tripartite co-culture with a 1:1 bacterial mixture (Fig. 2E), the PIs of both bacteria (12.4 ± 4.2 for wt and 13.7 ± 4.4 for *∆pys*) remained comparable (*P* = 0.95). The inter-ratio comparison (Fig. 2E) among tripartite co-cultures (with 1:3 and 3:1 bacterial mixtures) revealed that the PI of wt (5.1 ± 2.0) in the 1:3 ratio was comparable to the PI of *∆pys* (6.2 ± 2.3) in the 3:1 ratio. A similar trend could be observed between the PI of *∆pys* in the 1:3 ratio and the PI of wt in the 3:1 ratio (*P* = 0.35). The variation in the PIs of both wt and *∆pys* in the 1:3 ratio could be reversed in the co-culture with a 3:1 ratio of the bacteria (Fig. S2). Overall, these findings show that amoebae do not exhibit any feeding preference toward the producer or the mutant strain.

### Alterations in nutrient availability lead to diminished production of pyreudiones

Since *P. fluorescens* wt was found to be ingested by amoebae, the role of pyreudione A production on the temporal dynamics of this amoebae−bacteria interaction was investigated. We performed a time course analysis on the production of pyreudione A in co-cultures prepared in two media. First, in a nutrient-rich medium, PYG100 and second in a nutrient-poor medium, 10% of PYG100 medium (PYG10). Despite being a nutrient-poor medium, both amoebae and bacteria could still grow in PYG10 (Figs S4 and S5). The production of pyreudione A also occurred at lower titers in PYG10 when compared to PYG100 media (Fig. S3). Since bacterial virulence and other evasion mechanisms have been reported to be mediated by cell density and other population-dependent processes [58, 76, 77], we inoculated the bacteria with *D. discoideum* at a MOI of 5 or 100 bacteria per amoeba.

**Figure 3:**
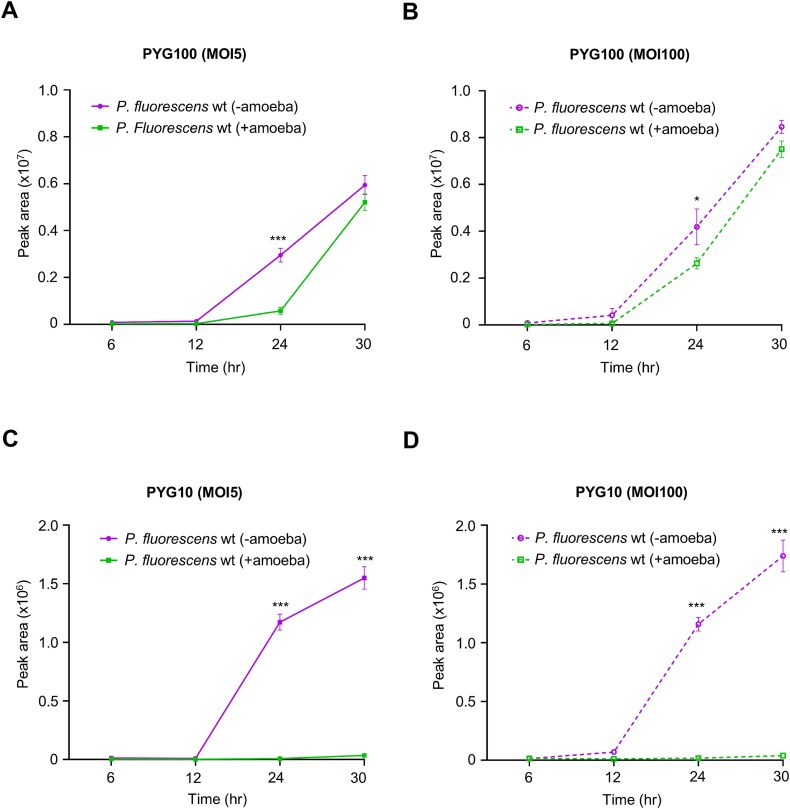
Production of pyreudione is diminished in co-cultures with nutrient-depleted media. (A) the production of pyreudione A in the co-culture prepared with PYG100 media and the bacterial population at MOI 5. (B) The production of pyreudione A in the co-culture prepared with PYG100 media and the bacterial population at MOI 100. The levels of pyreudione gradually increase over time in co-cultures prepared with PYG100 media. (C) The production of pyreudione in the co-culture prepared with PYG10 media and the bacterial population at MOI 5. (D) The production of pyreudione in the co-culture prepared with PYG10 media and the bacterial population at MOI 100. Significant reduction in the production of pyreudione can be observed over time in the co-cultures prepared with PYG10 media. The production of pyreudione at each time point is represented as the peak area of pyreudione A (at λ = 190 nm). Data shown are mean ± standard error combined from three independent experiments. A two-way ANOVA with Holm–Šídák’s multiple-comparisons test was used to determine the significance of the production of pyreudione a at each time point between monocultures and co-cultures (symbols: ^*^P ≤ 0.05, ^*^^*^^*^P ≤ 0.001).

In the PYG100 co-culture, the production of pyreudione A gradually increased with time at both inoculation densities: MOI 5 and 100 (Fig. 3A and B). At the final time point (30 h), the level of pyreudione A in the co-culture was comparable to the monoculture of *P. fluorescens* wt in PYG100 media (*P* = 0.13 for MOI 5 and *P* = 0.23 for MOI100).

Interestingly, in the co-culture with PYG10, at both inoculation densities, the production of pyreudione A was significantly diminished when compared to the monoculture of *P. fluorescens* wt in the same media (Fig. 3C and D). The pyreudione production in the monoculture of *P. fluorescens* wt at both MOIs gradually increased after 12 h. At the final time point, the production was significantly higher when compared to the co-cultures. The predation of bacteria from earlier time points could have caused the decline in the population of *P. fluorescens* wt and eventually led to diminished levels of pyreudione A.

This could also explain the lag in the production of pyreudiones in PYG100 media at an earlier time point (24 h) when compared to the monoculture (Fig. 3A). Because PYG100 is a rich medium, bacteria outgrew the amoebae and increased the levels of pyreudione A in the co-culture. These findings demonstrated the influence of nutrition availability in altering the antipredator defense mechanisms.

### Allee effect influences microbial predator–prey relationship

To identify a potential link between cell density and the overall fitness of *P. fluorescens* HKI0770, we inoculated the bacteria with *D. discoideum* at two MOIs (5 and 100). We then determined the amoebal cell density and performed a CFU count assay of the bacteria. The time-dependent changes in the cell density of amoebae and bacteria were recorded from the co-cultures prepared in PYG100 and PYG10 media.

**Figure 4:**
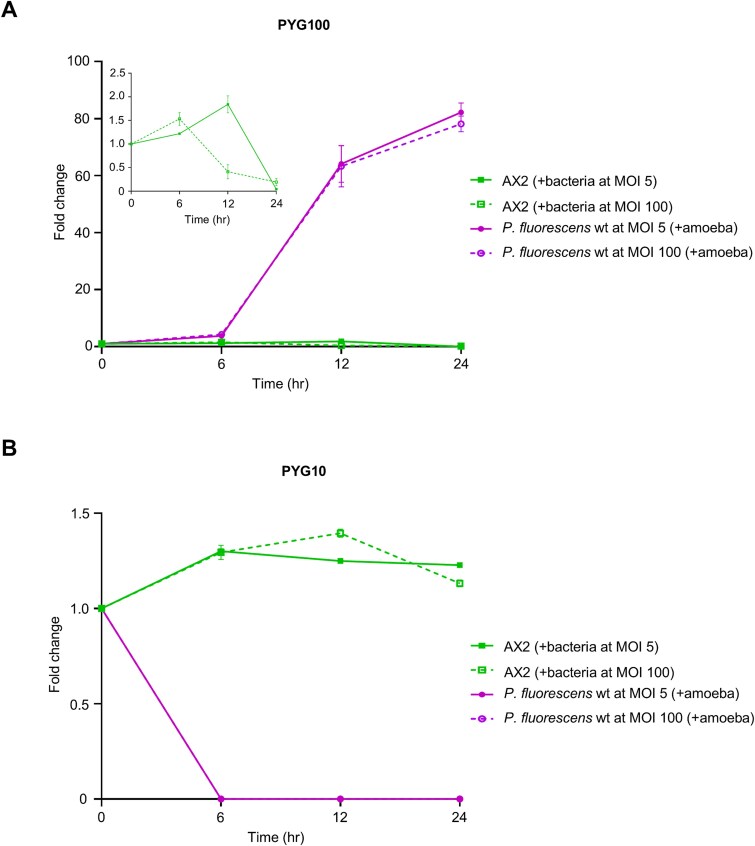
A strong Allee effect influences predator–prey relationship. (A) In PYG100 media, the bacteria population (at MOI 5 and 100) increases over time while there is a decline in the population of amoeba after 6 h. The differences in the growth of bacteria at both MOIs (5 and 100) were comparable. The inset shows an enlarged representation of the decline of the amoebal population in co-culture over time. (B) In PYG10 media, the population of amoeba shows a slight increase but rather remains stable throughout the timepoints. But there is a significant decline in the bacterial population starting from 6 h. The differences in the growth of amoeba at both MOIs (5 and 100) were not significant. The change in amoebae or bacterial population is represented as fold change (y-axis) over time (x-axis). Data shown are mean ± standard error combined from three independent experiments. Statistical significance was determined using two-way ANOVA with Holm–Šídák’s multiple-comparisons test by comparing the fold change of amoeba to the fold change of bacteria in the respective co-culture at each time point.

In the PYG100 co-culture, exponential growth of bacteria was observed at both inoculation densities: MOI 5 and 100 (Fig. 4A). Even though an initial increase in amoebae cell numbers was seen (Fig. 4A inset), a decrease occurred as the bacterial population attained exponential growth after 6 h. Together with the quantifications of pyreudione titers within the co-cultures (Figs 3A and B; S6), we concluded that bacterial growth in nutrient-rich PYG100 medium resulted in the production of pyreudiones leading to the decline of the amoebal population.

However, the co-culture in PYG10 medium displayed amoebal cell numbers that were rather stable throughout the duration of the assay at both inoculation densities, when compared to growth of bacteria at the respective MOI: MOI 5 (*P* ≤ 0.001,) and 100 (*P* ≤ 0.001; Fig. 4B). In contrast, there was a significant reduction in the bacterial population indicating that the amoeba was consuming bacteria (Figs 4B; 2A). At later time points, the bacterial population in co-culture was virtually depleted (fold change = 0 from 6 h at both MOIs) resulting in the absence of pyreudione A (Fig. 3C and D). Additionally, we could also demonstrate that amoebal secretions did not alter the growth of bacteria (*P* = 0.79 for normal and conditioned PYG100 media and *P* = 0.25 for normal and conditioned PYG10 media; Fig. S4), rather, predation by amoeba was the cause of the decline in bacterial population.

Alterations in nutrition and variations in the bacterial cell density determined the level of pyreudione A in the system and therefore facilitated the predation of *P. fluorescens* HKI0770. Hence, in this predator–prey relationship, both bacteria and amoebae exhibit a direct link between population size and their individual fitness. We propose that this feedback mechanism is the result of a strong Allee effect [49, 50, 78].

### Interplay between nutrient availability and a strong Allee effect

Even though the influence of Allee effects on amoebae−bacteria interactions has been elucidated [47, 48], the circumstances in which amoebae could overcome the potent evasion strategies of bacteria remain poorly understood.

To assess how nutrient availability coupled with a strong Allee effect determines the outcome of this predator–prey relationship, we analyzed the fruiting body formation of *D. discoideum* in co-culture with *P. fluorescens* wt. Three media (PYG100, PYG10, and PAS) with decreasing nutrient content and PAS being non-nutrient saline [79] were used. Different starting bacterial densities (MOI 5 and 100) were also tested.

**Figure 5:**
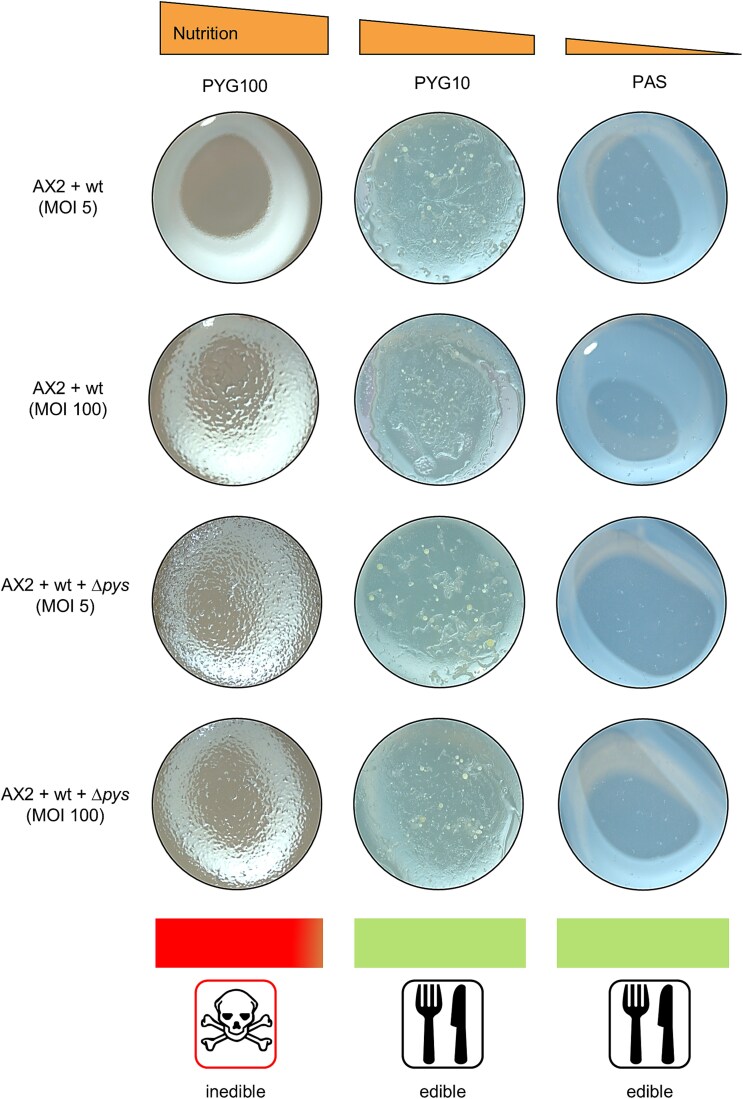
Interplay between nutrient availability and a strong Allee effect promotes the predation of *P. fluorescens* wt. Plaque assay with bipartite and tripartite co-cultures of amoeba and *P. fluorescens* HKI0770 strains. Fruiting bodies can be seen on the agar plugs as the nutrient availability decreases from left to right indicating predation by amoeba. Because PAS is a non-nutrient saline, the fruiting bodies on PAS agar are smaller than those in other media. The experiment was performed in two independent replicates.

In the wells with PYG100 media, the nutrient availability was sufficient for the bacteria to grow and compete with amoebal predation even at lower inoculation densities. This resulted in the production of pyreudiones and thereby killing the amoeba as described before [23]. As a result, no fruiting bodies were observed on these wells (Fig. 5). Having varying extracellular concentrations of pyreudione A in a co-culture can effectively alter the feeding behavior of amoebae (Fig. S7).

In the case of wells with nutrient-poor PYG10 and PAS, fruiting bodies of *D. discoideum* could be observed in the bipartite and tripartite co-cultures (Fig. 5). In nutrient-poor media, bacterial growth did not reach exponential rates as the amoebae were able to feed on the bacteria and exert predation pressure (Fig. 5). The bacterial population did not reach the critical threshold required to produce enough pyreudiones to kill the amoebae (Fig. 3C and D). Thus, creating a strong Allee effect on the bacteria which eventually leads to the decline of the bacterial population. With no bacteria remaining, amoebae started to aggregate and form fruiting bodies. Therefore, nutrient-depleted conditions and diminished pyreudione production provide a favorable environment for amoebal predation (Figs 2A; 3C and D; 4B; 5).

### Amoebal grazing alters the bacterial distribution

Predation of bacteria by protists (including amoebae) has been shown to cause changes in bacterial distribution and diversity [11, 16, 80]. To understand the interplay between amoebal predation and nutrient availability, we established a dual−agar system that allows monitoring predation while varying nutrient availability as encountered in many natural habitats. Two combinations of dual−agar systems (PYG100–PYG10 and PYG10–PAS) were prepared.

**Figure 6:**
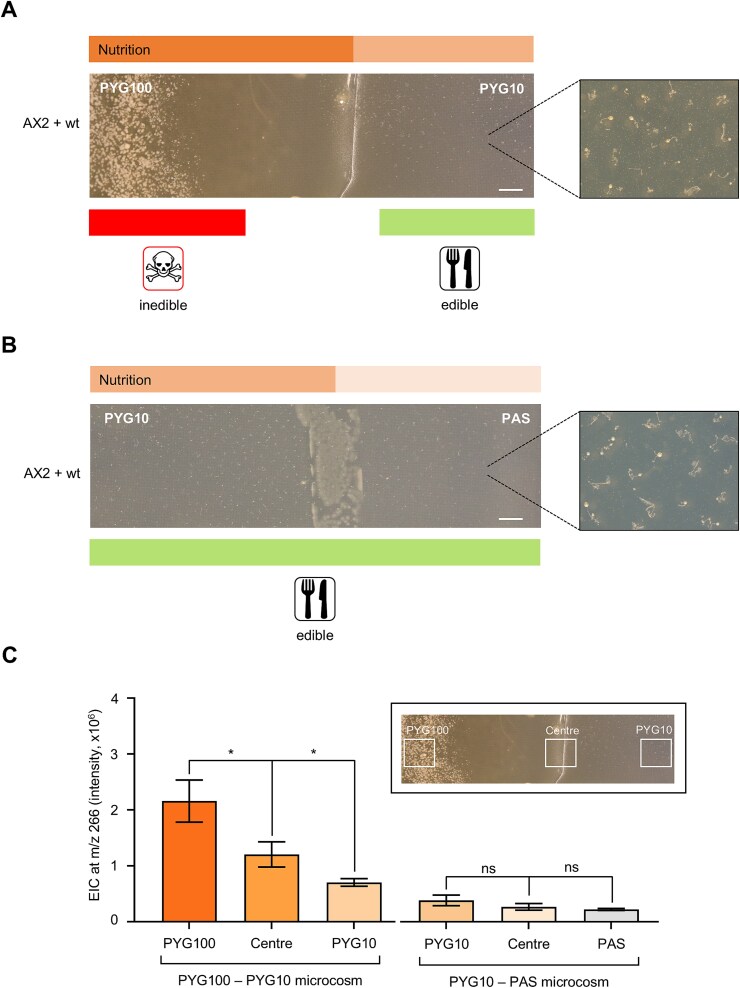
Co-culture of *D. discoideum* AX2 and *P. fluorescens* wt on dual–agar microcosm. (A) In the PYG100–PYG10 microcosm, bacterial colonies can be seen toward the nutrient-rich (left) side of the microcosm. Fruiting bodies are spread throughout the rest of the microcosm. (B) In the PYG10–PAS microcosm, fruiting bodies can be seen spread throughout both media. Insets show an enlarged view of the microcosm with the fruiting bodies. The experiment was performed in three independent replicates. (C) Comparison of the EIC values of pyreudione a (m/z 266) from different parts of the microcosms (PYG100–PYG10 and PYG10–PAS). The inset shows a representative image of the dual–agar microcosm with white squares indicating the areas from which agar pieces are obtained for UHPLC–MS. Data shown are mean ± standard error combined from three independent experiments. An ordinary one-way ANOVA with Holm–Šídák’s multiple-comparisons test was applied to determine the statistical significance of the distribution pyreudione A across the dual–agar microcosm (symbols: ns non-significant, ^*^P ≤ 0.05). Scale bars are 0.5 cm.

In the PYG100–PYG10 dual−agar system, bacterial colonies were more abundant on the nutrient-rich (PYG100) part of the microcosm without any signs of fruiting body formation (Figs 6A; S8A). The remainder of the microcosm was, however, filled with fruiting bodies indicating bacterial consumption. This was further reflected by the distribution of pyreudione A in the microcosm (Fig. 6C). The nutrient-rich (PYG100) section of the microcosm had a higher concentration of pyreudione A (peak area = 2.1 × 10^6^ ± 3.76 × 10^5^) when compared to the center section (peak area = 1.2 × 10^6^ ± 2.24 × 10^5^; *P* = 0.03 for PYG100 vs Centre) and nutrient-poor (PYG10) section (peak area = 0.7 × 10^6^ ± 0.67 × 10^6^; *P* = 0.01 for PYG100 vs PYG10) of the microcosm.

**Figure 7:**
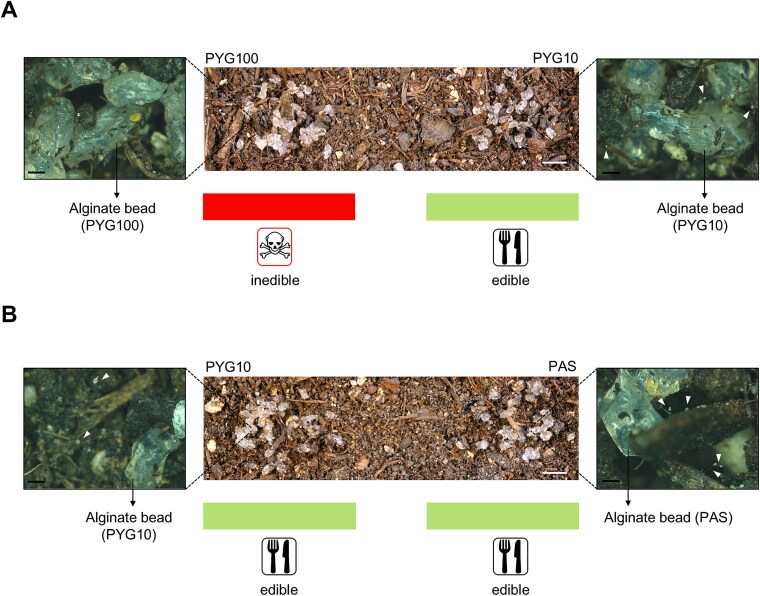
Co-culture of *D. discoideum* AX2 and *P. fluorescens* wt on soil–alginate hydrogel microcosm. (A) In the PYG100–PYG10 soil microcosm, no fruiting bodies can be seen on the nutrient-rich part (PYG100) of the microcosm. But fruiting bodies are spread throughout the nutrient-poor part (PYG10) of the microcosm–indicating amoebal predation on the bacteria. (B) In the PYG10–PAS microcosm, fruiting bodies can be seen spread on both halves of the microcosm. Insets show an enlarged view of different halves of the microcosm with alginate beads. White arrowheads indicate fruiting bodies formed on alginate beads and soil particles in the microcosm. The experiment was performed in two independent replicates. Scale bars are 0.5 mm (black) and 0.5 cm (white).

**Figure 8:**
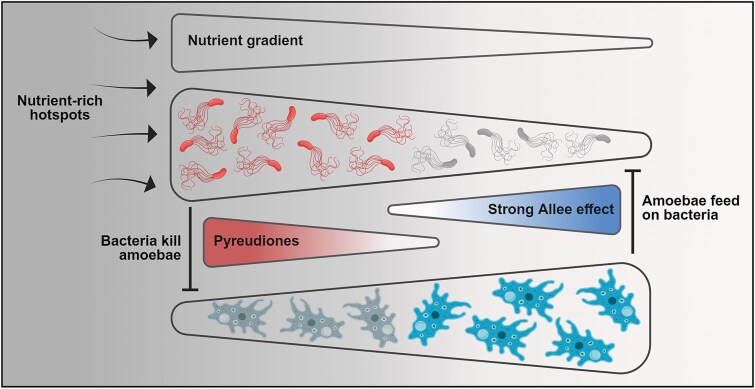
A schematic representation of the amoeba−bacteria interaction discussed in this study. In nutrient-rich hotspots, the enhanced nutrient availability provides the right ecological niche for the soil-dwelling bacteria, *P. fluorescens* HKI0770 to multiply and secrete pyreudiones. As a result, bacterivorous organisms such as amoebae are unable to feed on the bacteria. As the bacteria move away from such hotspots, the nutrient availability and the cell density tend to decrease. Thereby creating a strong Allee effect and resulting in the diminished secretion of toxic natural products. Thus, rendering *P. fluorescens* HKI0770 vulnerable to free-living protists. Further highlighting the role of pleiotropic regulation in microbial predator–prey dynamics. The illustration was created with BioRender.com.

Bacterial proliferation is higher in the nutrient-rich part of the microcosm, leading to higher concentrations of pyreudione A resulting in the death of the amoeba. Lack of proper nutrition and predation pressure by amoeba resulted in the decline of the *P. fluorescens* wt population in the nutrient-poor section of the microcosm.

The microcosm composed of two nutrient-poor media (PYG10 and PAS) showed a different outcome. The presence of fruiting bodies across both media indicates that the bacteria were unable to produce enough pyreudione A to defend themselves against predation (Figs 6B; S8B). The abundance of pyreudione A in different parts across the PYG10–PAS microcosm was also comparable (Fig. 6C; *P* = 0.40 for PYG10 *vs* Centre and *P* = 0.21 for PYG10 *vs* PAS). Even though bacteria secrete low amounts of pyreudione A in PYG10 media (Fig. S3), the lack of proper nutrition and a strong Allee effect generated by amoebal predation resulted in the decline of the *P. fluorescens* wt.

Protists such as Dictyostelids are common inhabitants of most soils, where they can graze on Gram-negative bacteria such as Pseudomonads [80, 81]. To investigate such perturbations commonly encountered by soil bacteria, we established soil microcosms based on the principle of the dual−agar system. Alginate beads added to the microcosm promote the slow release of nutrition into the soil while maintaining moisture throughout the experiment [82, 83]. In the PYG100–PYG10 soil–alginate microcosm, fruiting bodies could be seen on the nutrient-poor (PYG10) part of the microcosm, however the absence of fruiting bodies can be observed on the nutrient-rich part (PYG100) of the microcosm. Similar to the PYG100–PYG10 dual−agar system, bacteria in the nutrient-rich part were able to produce pyreudione A and kill the amoebae; simultaneously amoebal predation occurred in the nutrient-poor part of the microcosm.

On the contrary, the soil–alginate microcosm composed of alginate beads made using two nutrient-poor media (PYG10 and PAS) showed the presence of fruiting bodies across both media indicating the occurrence of amoebal grazing as a result of the absence of pyreudione A; which is *on par* with the results in PYG10 – PAS dual−agar system (Fig. 7). Hence, our models emphasize the critical role of nutrient availability and population density in shaping microbial predator–prey relationships prevalent in various ecological niches.

## Discussion

Among various social interactions, predation exerts a strong selection pressure on bacteria. The secretion of anti-predator natural products by bacteria is a potent countermeasure [11, 58]. Although these bacteria are classified as extracellular pathogens to amoebae and collectively evade amoebal predation [23, 84], the interaction on a microscopic scale between individual amoebae and bacteria remains poorly understood. Our results revealed that under certain conditions the soil-dwelling bacterium *P. fluorescens* HKI0770, known for producing amoebicidal natural products and previously shown to evade predation on a macroscopic scale, was, in fact, ingested by the amoeba.

Prey selection plays a critical role among the foraging strategies employed by amoebae as the nature of prey (bacteria) directly influences their fitness [21, 25]. Studies have shown that amoebae have different mechanisms to sense and recognize bacteria [22, 85, 86]. However, our knowledge about the mechanisms of selective grazing in amoebae remains limited. Feeding experiments involving bacterivores showed that amoebae such as *Acanthamoeba castellanii* exhibit preferential feeding, even discriminating isogenic bacterial strains [87, 88]. However, our investigation into the ingestion of amoebicidal bacteria suggests that *D. discoideum* was not able to distinguish between the pyreudione producer strain and the non-producer mutant (*∆pys*) indicated by similar phagocytic indices—further highlighting the critical role of toxic small molecules in determining the feeding behavior of amoebae [23, 24, 36]. The underlying differences in the recognition mechanisms across the major clades of amoebae could explain the variability in the foraging strategies among amoebal species [89, 90]. This further emphasizes the abundance and diversity of protists as a powerful, yet necessary force in shaping natural microbial communities.

Interestingly, our findings revealed that the circumstances leading to the phagocytosis of amoebicidal bacteria were closely linked to nutrient availability and the presence of a strong Allee effect. Such cell density-dependent phenomena emerge when the population levels fall below a critical threshold [48]. Time-dependent co-culture studies offered insights into this hypothesis as well as the temporal dynamics of the amoebae−bacteria interaction. Notably, changes in nutrient availability and a strong Allee effect within the co-culture resulted in the diminished production of pyreudione A, thereby making *P. fluorescens* HKI0770 susceptible to amoebal predation. Since it is currently virtually impossible to retrace the complex microbial interactions and metabolic exchange within natural settings such as the rhizosphere [91, 92], our study allowed to shine light on such a microbial interaction in a laboratory setting.

Using our microcosm experiments (both agar- and soil-based microcosms), we were able to replicate these phenomena under laboratory conditions in which microbial predator–prey dynamics are influenced by population size and nutrient distribution (Fig. 8). In nutrient-rich hotspots, increased nutrient availability promotes growth and secretion of anti-predator natural products of soil-dwelling bacteria including *P. fluorescens* HKI0770. As a result, bacterivores are unable to feed on these pathogenic bacteria. However, as the bacteria move away from such hotspots, the nutrient availability and the microbial density decrease. This results in the diminished secretion of toxic natural products, rendering *P. fluorescens* HKI0770 vulnerable to free-living protists. We acknowledge that our microcosm experiments might not reflect structural heterogeneity and multispecies interactions that often prevail in natural habitats. However, our study clearly demonstrated the multifaceted nature of predator–prey interactions and the influence of environmental conditioning in shaping them.

It has been reported that upon predation pressure, not all amoebae cells aggregate to form fruiting bodies. Rather, some cells remain solitary, allowing them to migrate [93]. Similarly, changes in various biotic or abiotic factors can lead to incomplete consumption of bacteria, allowing some to survive predation and continue multiplying [94]. Intriguingly, the integration of such auxiliary processes into the existing microbial loop could further explain how bacterivores are able to feed on inedible bacteria through environmental conditioning and pleiotropic regulation of biosynthetic pathways. Therefore, these findings also shed light on the often-underestimated roles of protists in affecting the structure and function of microbial communities, with broader implications for microbiome engineering.

Investigating the diverse interactions between eukaryotes and bacteria can unravel new insights into the dynamics of interspecies relationships. However, deciphering such complex interactions across scales and multicellular hosts with varying bacterial diversity presents significant challenges. In contrast, understanding such interactions in a simplified system, with only a few interacting partners could unveil features of eukaryote–bacteria interactions that can be further expanded to complex microbiomes by adding different layers of complexity.

Taken together, in this study we have provided a direct experimental demonstration on the pleiotropic control of the production of natural products by bacteria could alter the feeding behavior of amoeba, turning inedible bacteria into edible ones and *vice versa*. Non-genetic regulation is crucial as it governs the outcome of, not only predator–prey interactions but the whole spectrum of symbiosis. Our findings significantly contribute toward understanding the mechanisms underlying the predation-evasion strategies of bacteria and how amoebae can form various symbiotic associations with bacteria.

Further, the application of new methodologies such as the chromatic toolbox for intraspecific differentiation in intricate symbiotic relationships such as predator–prey interaction is a novel strategy and paves the way for potential applications in microbial identification and host–microbe interactions. The mechanisms of pleiotropic regulation also demonstrate how environmental conditioning can influence the existing ecological processes, prompting questions regarding the synergy between pleiotropic and genetic regulation in shaping microbial communities.

## Supplementary Material

2_ISME_Comm_SI_11-02-2025_ycaf031

## Data Availability

All data reported in this paper are available from the corresponding author upon request.
