# Supplementary material for: Pleiotropic regulation of bacterial toxin production and Allee effect govern microbial predator–prey interactions
Source: ISME Commun. 2025 Feb 14;5(1):ycaf031. doi: 10.1093/ismeco/ycaf031 (PMC11904905; doi:10.1093/ismeco/ycaf031)
Supplement: 2_ISME_Comm_SI_11-02-2025_ycaf031 [file 2_isme_comm_si_11-02-2025_ycaf031.pdf]

Supplementary Information

Pleiotropic regulation of bacterial toxin production and Allee effect govern microbial predator–prey interactions

Harikumar R. Suma[1,2] and Pierre Stallforth[1,3,4*]

[1]Department of Paleobiotechnology, Leibniz Institute for Natural Product Research and Infection Biology – Leibniz-HKI, Beutenbergstrasse 11a, 07745 Jena, Germany

[2]Cluster of Excellence Balance of the Microverse, Friedrich Schiller University Jena, Jena, Germany

[3]Faculty of Chemistry and Earth Sciences, Friedrich Schiller University (FSU), Jena, Germany

[*]Correspondence: pierre.stallforth@leibniz-hki.de (P.S.)

Figure S1. Pyreudione production of *P. fluorescens* HKI0770 chromatic strains. (A) Plaque assay with *Dictyostelium discoideum* AX2 and *P. fluorescens* HKI0770 strains. (B) Comparison of stacked chromatograms between the chromatic mutant and *P. fluorescens* wt. The phenotypes of the chromatic mutants remain identical to their parental strains. Black arrowhead indicates the peak for Pyreudione A (UV detection was at λ = 190 nm).

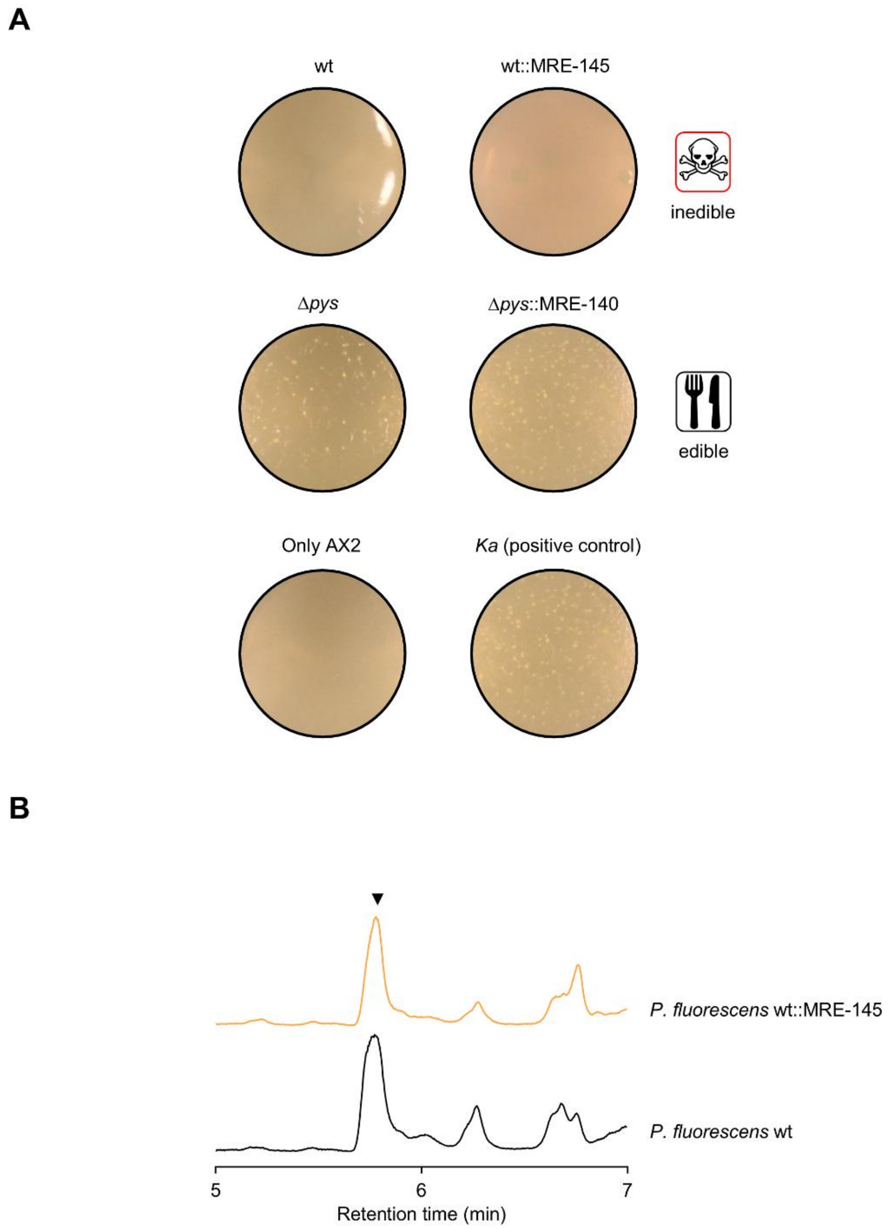

Figure S2. Comparison of the phagocytic indices among the co-culture. Quantification of phagocytic indices of wt and Δ*pys* strains in the different co-culture ratios (1:3, 3:1) based on the data shown in Fig. 2E. A two-way ANOVA with Holm-Šídák's multiple-comparisons test was performed to evaluate the statistical significance between the phagocytic indices of wt and Δ*pys* strains (symbols: ns not significant, * P ≤ 0.05, ** P ≤ 0.01).

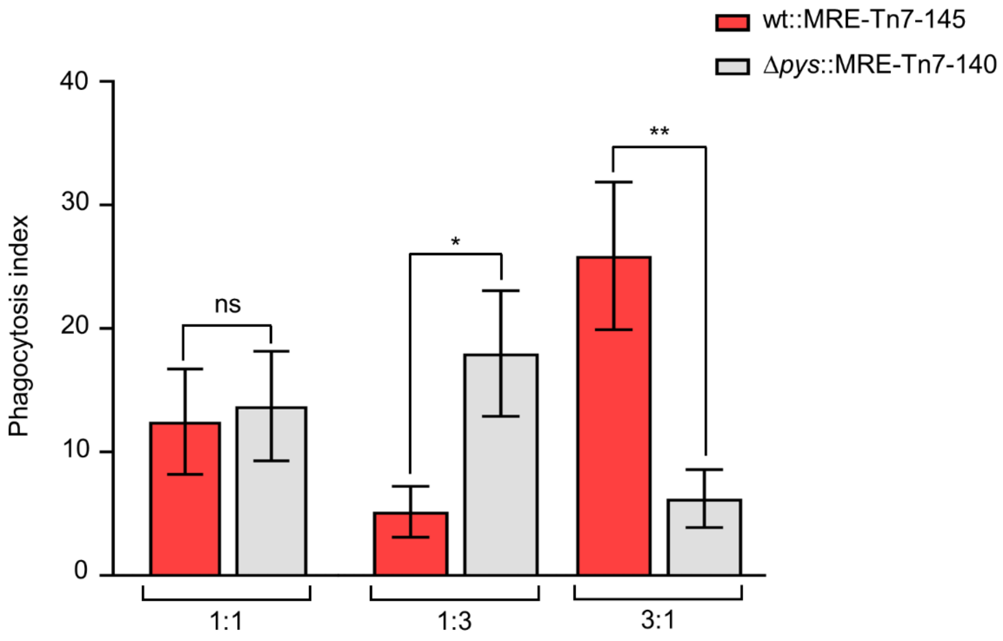

Figure S3. Production of pyreudione A by *P. fluorescens* wt in different media. (A) Stacked chromatograms showing the comparison pyreudione A production in PYG100, PYG10 and PAS. Black arrowheads indicate the peak for pyreudione A (UV detection at λ = 190 nm). (B) Production titre of pyreudione A in PYG100, PYG10 and PAS. Data shown are mean ± standard error combined from three independent experiments (n=3 for each media). Ordinary one-way ANOVA with Holm-Šídák's multiple-comparisons test was applied to determine the statistical significance (symbols: *** P ≤ 0. 0.001).

A

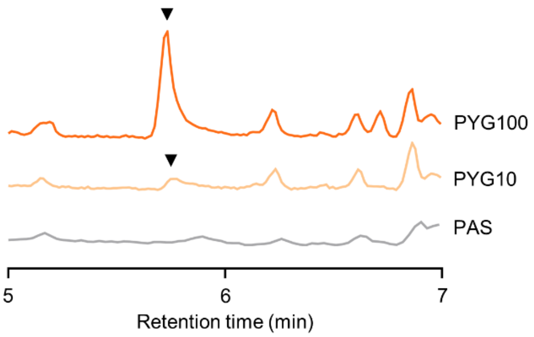

B

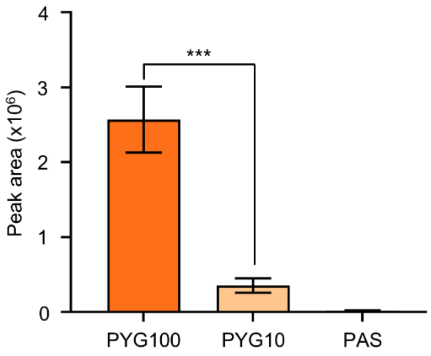

Figure S4. Growth of *P. fluorescens* wt in amoeba-conditioned media (ACM). The growth of *P. fluorescens* wt was comparable when grown in PYG100 and in PYG100 (ACM). A similar trend could be seen between PYG10 and PYG10 (ACM). Data shown are mean ± standard error (n=5 for each media). The experiment was performed in two independent experiments.

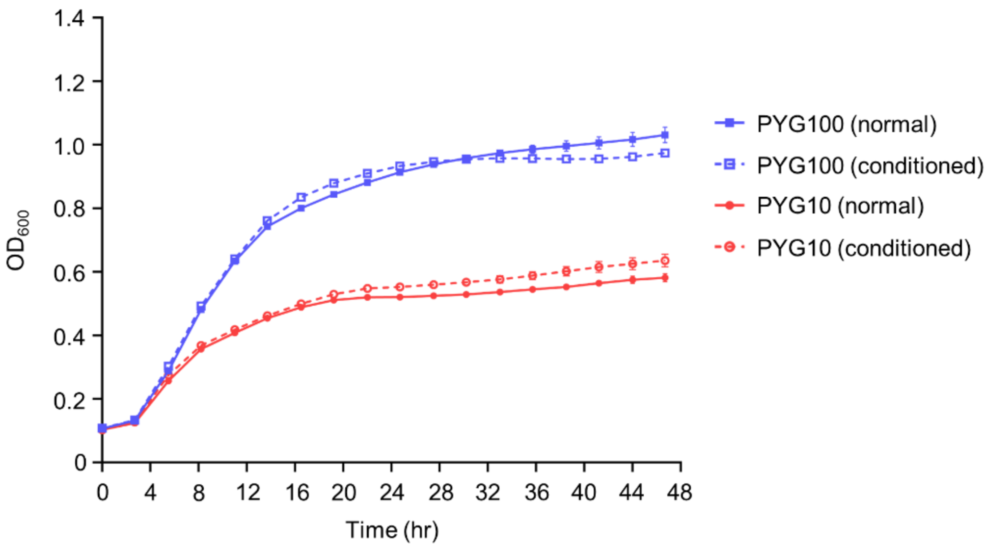

Figure S5. Growth of AX2 in different media.

Axenic growth of *D. discoideum* AX2 in HL5, PYG100 and PYG10 media. The increase in cell density represented as fold change over time. Data shown are mean ± standard error pooled from three independent experiments. Statistical significance was determined using two-way ANOVA followed by Holm-Šídák's multiple-comparisons test at each time point.

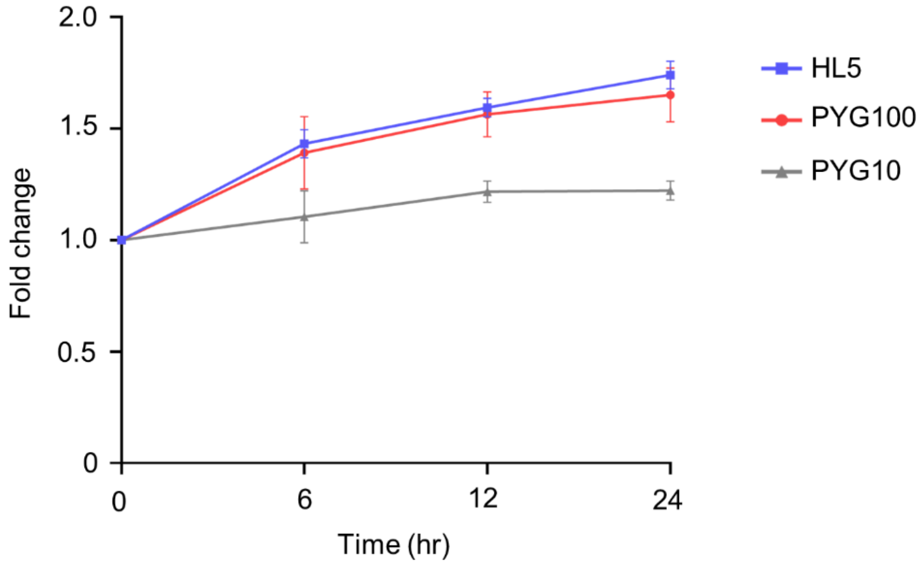

Figure S6. Production of pyreudione A in response to nutrient availability and cell density.

The production of pyreudione A in co-culture (MOI 5 and MOI 100) prepared with PYG100 and PYG10 media. The concentration of pyreudione A gradually increases over time in co-cultures prepared with PYG100 media whereas the production is diminished in PYG10 media. Data shown are mean ± standard error combined from three independent experiments. Statistical significance was determined using two-way ANOVA with Holm-Šídák's multiple-comparisons test by comparing the concentrations of pyreudione A in each co-culture at the respective time point (symbols: * $P \leq 0.05$, ** $P \leq 0.01$).

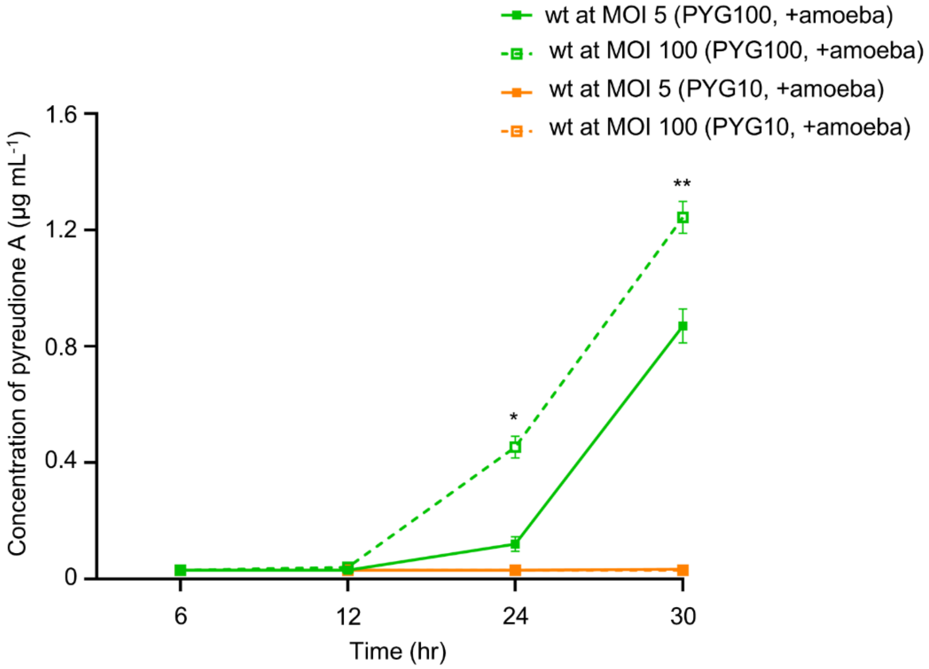

Figure S7. Co-culture of AX2 and ∆*pys* supplemented with pyreudione A. Plaque assay with co-cultures of amoeba and *P. fluorescens* ∆*pys*. Presence of pyreudione A at different concentrations in the co-culture kills the amoebae. But fruiting bodies can be observed at a lower concentration (0.03 and 0.06 µg mL^{-1}), indicating predation by amoebae. Further highlighting the influence of pyreudione A on amoebal predation. The experiment was performed in two independent experiments.

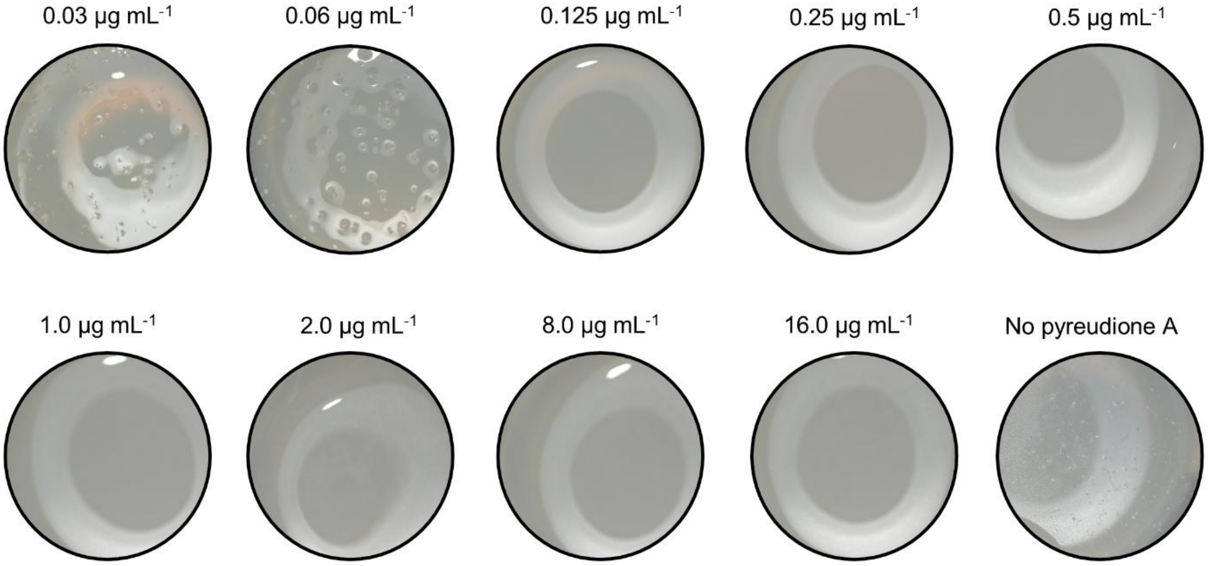

Figure S8. Co-culture of *D. discoideum* AX2 and *P. fluorescens* wt::MRE-145 on dual–agar microcosm.

(A) In the PYG100 – PYG10 microcosm, fluorescent (orange) bacterial colonies can be seen distributed towards nutrient-rich (PYG100) left side of the microcosm. Absence of bacterial colonies on the rest of the microcosm indicate amoebal predation on the bacteria. (B) In the PYG10 – PAS microcosm, an absence of fluorescence and distribution of fruiting bodies can be seen throughout both media.

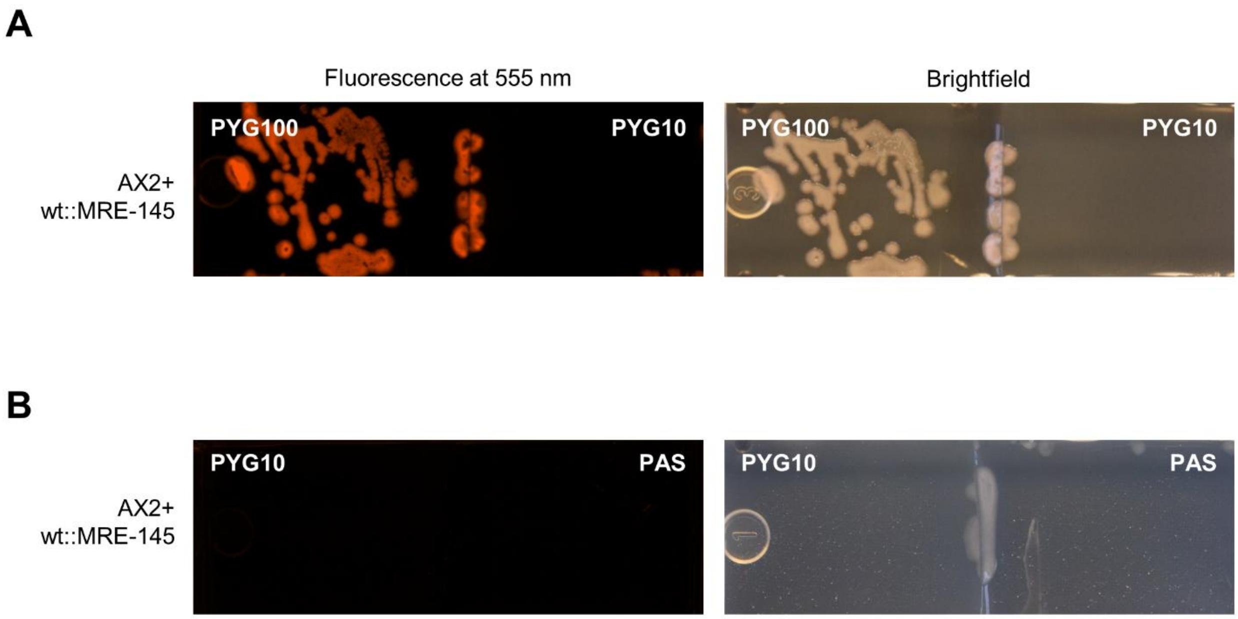

Table S1. List of all primer sets used in this study.

Gene Name	Forward Primer	Reverse primer	Classification
mScarlet-I, mTagBFP2	AAACTGGATGGCTTTCTTGC	CAACAGGAGTCCAAGCTCAG	Fluorescent tags
TnsABC+D-transposase complex	GGGCTTTTCTAGTTCTGACA	AGTGCTGGTTGATACGATTT	pMRE-Tn7 plasmid backbone
